# Balancing adaptability and standardisation: insights from 27 routinely implemented ICHOM standard sets

**DOI:** 10.1186/s12913-022-08694-9

**Published:** 2022-11-28

**Authors:** Leo Benning, Zofia Das-Gupta, Luz Sousa Fialho, Stephanie Wissig, Neo Tapela, Suzanne Gaunt

**Affiliations:** 1 University Emergency Center, Medical Center – University of Freiburg, Freiburg, Germany; 2 Faculty of Medicine, University of Freiburg, Freiburg, Germany; 3 International Consortium for Health Outcomes Measurement, London, UK; 4 International Consortium for Health Outcomes Measurement, Boston, USA

**Keywords:** Outcomes, Patient-reported, Value-based health care

## Abstract

**Background:**

Healthcare systems around the world experience increasing pressure to control future growth of healthcare expenditures. Among other initiatives, quality and value-based benchmarking has become an important field to inform clinical evaluation and reimbursement questions. The International Consortium for Health Outcomes Measurement (ICHOM) has become one of the driving forces to translate scientific evidence into standardized assessments that are routinely applicable in day-to-day care settings. These aim to provide a benchmarking tool that allows the comparison and competition of health care delivery on the basis of value-based health care principles.

**Methods:**

This work focuses on the consolidation of the ICHOM methodology and presents insights from 27 routinely implemented Standard Sets. The analysis is based on a literature review of the ICHOM literature repository, a process document review and key informant interviews with ICHOM’s outcomes research and development team.

**Results:**

Key findings are that the scope of ICHOM Standard Sets shifted from a more static focus on burden of disease and poorly standardized care pathways to a more dynamic approach that also takes into account questions about the setting of care, feasibility of implementing a benchmarking tool and compatibility of different Standard Sets. Although certain overlaps exist with other initiatives in the field of patient reported outcomes (PRO), their scopes differ significantly and they hence rather complement each other. ICHOM pursues a pragmatic approach to enable the benchmarking and the analysis of healthcare delivery following the principles of value-based healthcare.

**Conclusion:**

The ICHOM Standard Sets complement other initiatives in the field of patient-reported outcomes (PRO) and functional reporting by placing a particular focus on healthcare delivery, while other initiatives primarily focus on evaluation of academic endpoints. Although ICHOM promotes a pragmatic approach towards developing and devising its Standard Sets, the definition of standardized decision making processes emerged as one of the key challenges. Furthermore, the consolidation of core metrics across number of disease areas to enable the parallel implementation of different Standard Sets in the same care setting is an important goal that will enable the widespread implementation of patient-reported outcome measures (PROM).

## Introduction

Healthcare expenditures have been exceeding real growth of GDP for decades and more and more healthcare systems are under pressure to control further growth of their spending [1]. The U.S. reached $ 3.8 trillion in healthcare spending in 2019, another substantial increase of 4.6% from the previous year, and healthcare spending now accounts for 17.7% of the total GDP [2]. Although the U.S. is not alone in confronting this challenge [3], its annual per-capita spending is currently almost twice that of other high-income countries [4]. Despite high healthcare expenditures, the U.S. performs poorly on key health performance indicators such as life expectancy, preventable and amenable mortality, and population coverage for a core set of services [5].

Given this backdrop, the U.S. government has been reorienting its approach towards care delivery and now emphasizes quality and value as new core metrics used to generate evidence, determine reimbursement and empower patients to make informed decisions about healthcare service utilisation. While early efforts for the introduction of performance-based payment systems date back to the 1990s [6, 7], major legislative efforts (i.e. the Affordable Care Act and the Medicare Access and CHIP Reauthorization Act) are now greatly promoting the incorporation of quality- and value-based principles into research [8] and reimbursement schemes [9], which have moved the aspects of patient-centered outcome (PRO) reporting, quality and value up on the public agenda. How best to deliver, and measure delivery of, value-based healthcare remains greatly debated.

In this evolving field, the International Consortium for Health Outcomes Measurement (ICHOM) has emerged as one of the organisations advancing value-based healthcare, with a particular emphasis on patient-centered outcome measurement and quality. ICHOM’s mission is rooted in Porter’s and Teisberg’s seminal work on value-based healthcare [10], which defines value as improvement in health outcomes per dollar spent. It proposes to shift competition from zero-sum (i.e. any advantage that is gained by one party is lost by another party) to increased value for patients [11, 12]. ICHOM’s work driven by this mission includes the development of condition-specific measures of health outcome (Standard Sets), their respective validation, and the facilitation of the implementation of these Standard Sets. These efforts aim towards fostering benchmarking and collaborative learning between healthcare contexts on a national and global level. Since its founding in 2012, ICHOM has developed 40 Standard Sets in consultation with more than 900 experts and, along with partners, has supported implementation in selected health care settings in 44 countries [13]. In total, the Standard Sets available cover almost 50% of the global burden of disease [14]. This article focuses on the articles that have been published as the primary reports about the respective Standard Sets, examines the spectrum of conditions covered and consolidates the methodology applied.

## Methodology

**Fig. 1:**
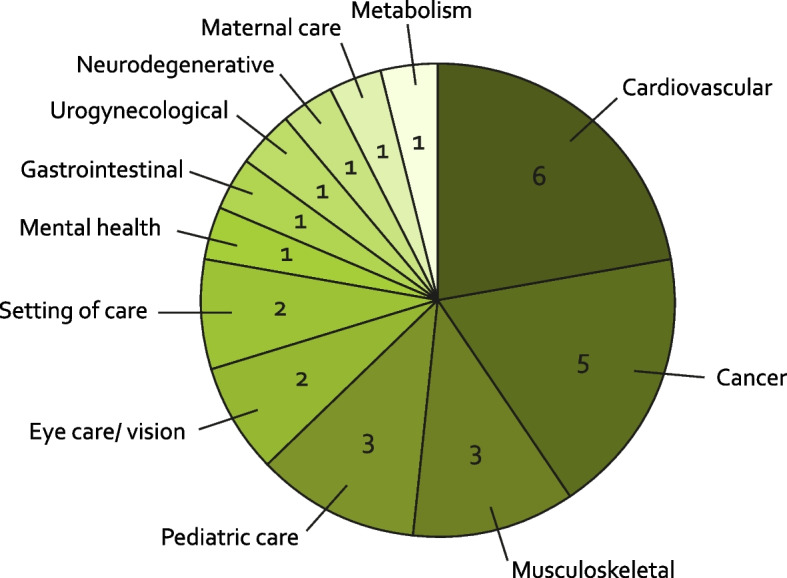
Distribution of conditions addressed by published ICHOM Standard Sets

This was a review of published literature describing development of ICHOM Standard Sets. Of the 40 available ICHOM Standard Sets, 27 have associated publications in peer-reviewed journals which describe the Sets development process (Table 1, Fig. 1). The remaining 13 Standard Sets are, at the time of our literature research, in the process of publication. Table 1 lists the names of the 27 Standard Sets for which publications were reviewed. Publications were from 2015 to 2021. The publications reviewed were identified using ICHOM’s online repository ‘Connect’, and through consultation with ICHOM’s outcomes research and development team. The first author read the articles in full and collated findings regarding scope, methodology, consensus finding process, domains covered and measures used. All findings were discussed with members of ICHOM’s team and reviewed in context of all published Standard Sets.

**Table 1:** Peer-reviewed ICHOM Standard Sets, conditions addressed, disease spectrum and estimated burden of disease

Authors	Condition	Category
Morgans et al. [15]	PCA, advanced	Cancer
Martin et al. [16]	PCA, localized	Cancer
Mahmud et al. [17]	Cataract surgery	Eye care/vision
McNamara et al. [18]	CAD	Cardiovascular
Clement et al. [19]	Lower back pain	Musculoskeletal
Ong et al. [20]	BCA	Cancer
Allori et al. [21]	Cleft care	Pediatric care
Rolfson et al. [22]	OA, hip/knee	Musculoskeletal
Mak et al. [23]	NSCLC/SCLC	Cancer
Rodrigues et al. [24]	AMD	Eye care/vision
Salinas et al. [25]	Stroke	Cardiovascular
Zerillo et al. [26]	CRC	Cancer
Obbarius et al. [27]	Depression, anxiety; adult	Mental health
Kim et al. [28]	IBD	Gastrointestinal
Foust-Wright et al. [29]	OAB	Urogynecological
De Roos et al. [30]	Parkinson’s disease	Neurodegenerative
Verberne et al. [31]	CKD	Cardiovascular
Akpan et al. [32]	Older Persons	Setting of care
Nijagal et al. [33]	Pregnancy/childbirth	Maternal care
Burns et al. [34]	HF	Cardiovascular
Zack et al. [35]	aHT	Cardiovascular
Voshaar et al. [36]	Inflammatory Arthritis	Musculoskeletal
Seligman et al. [37]	AFib	Cardiovascular
Nano et al. [38]	Diabetes	Metabolism
Algurén et al. [39]	Pediatric Health	Pediatric care
Ni Riordain et al. [40]	Oral health; adult	Setting of care
Krause et al. [41]	Depression, anxiety, OCD, PTSD; childhood/youth	Pediatric care

*PCA* Prostate cancer, *CAD* Coronary artery disease, *BCA* Breast Cancer, *OA* Osteoarthritis, *NSCLC* Non-small cell lung cancer, *SCLC* Small cell lung cancer, *AMD* Age-related macular degeneration, *CRC* Colorectal carcinoma, *IBD* Inflammatory bowel disease, *OAB* Overactive bladder, *HF* Heart failure, *aHT* Arterial hypertension, *AFib* Atrial fibrillation, *OCD* Obsessive-compulsive disorder, *PTSD* Post-traumatic stress disorder

## Results

### The spectrum of available ICHOM standard sets

The published available 27 ICHOM Standard Sets cover a vast range of the global burden of disease and address 12 of the 25 leading causes of disability globally in 2019 [42]. In addition to covering high burden conditions, we also observed that a subset of Standard Sets focused on conditions that represented challenges with managing them. Examples include the Standard Sets for overactive bladder syndrome (OAB) [29], care in the context of a cleft palate [21] and overall oral health in adults [40]. Additionally, some Standard Sets do not address medical conditions in the narrow sense, but rather focus on living status and wellbeing across the life course. Examples are Older Persons [32], overall Pediatric Health [39], and Pregnancy and Childbirth [33]. One Standard Set focuses on the highly prevalent condition of hypertension, but with a particular focus of measurement and care appraisal in low and middle income countries (LMIC) [35].

Nonetheless, ICHOM Standard Sets are developed from an international perspective and their wide applicability is generally a distinctive feature that sets ICHOM Standard Sets apart from other initiatives in the field of PRO and functional outcome reporting, which often focus on a narrow group of conditions [43–45]. Yet, the broad scope and a decentralized process of Standard Set development require a flexible methodological approach that allows adaptation to the clinical setting of each condition of interest.

### The process of the ICHOM standard set development

**Fig. 2:**
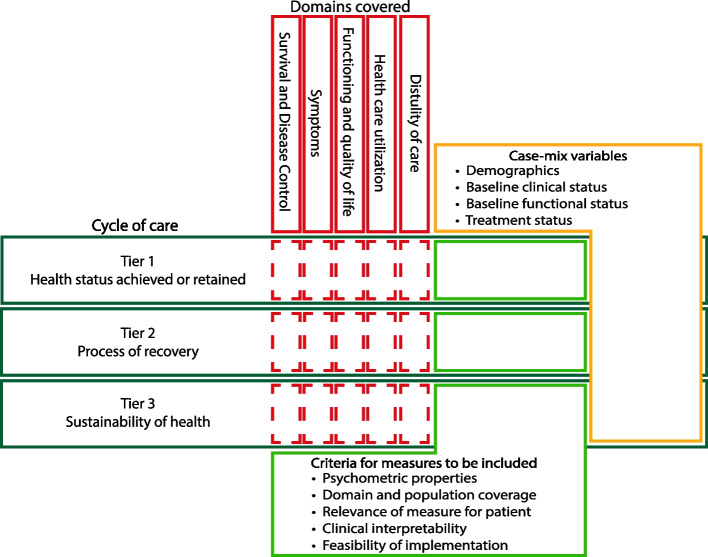
Integrated framework for the composition of an ICHOM Standard Set. The cycle of care concept (black) is adapted from Porter [46]. Domains are generic (red) and span across the entire cycle of care, but are not necessarily included in all tiers (dashed). Measures (green) are selected as they are considered to meet the criteria for inclusion. The decision for inclusion or exclusion into a Standard Set is based on the Delphi process outlined below. Case-mix variables (yellow) are included to allow for comparisons between sub-groups and stratifications

Key steps of the development are illustrated in Fig. 2. The methods of development for each Set are outlined in detail in the referenced publications included in Table 1. Below, we summarise these steps highlighting patterns in the development process across Standard Sets and over time.

#### Initiation of a standard set development project

Since the establishment of ICHOM, the decision to develop a specific Standard Set has primarily been driven to address conditions with a particularly high burden of disease and, secondarily, those with poorly standardized clinical management for the given condition (Table 1). In this context, the available evidence suggests that a structured framework for capturing patient-centered outcomes in clinical practice may reduce costs and drive up quality of care and would hence result in a higher healthcare value [47], which addresses the very principle of VBHC, as outlined before [12, 46].

ICHOM has leveraged its stakeholder network to monitor conditions and clinical care settings for which a new Standard Set might be beneficial. Initially more statically focused on disease prevalence and variability in quality and care delivery, the selection of a condition has evolved to a more dynamic shared decision-making process between medical experts with experience in the field of VBHC, the burden of disease and the feasibility of devising a comprehensive, yet condensed Standard Set for the respective condition.

After selecting an appropriate condition, ICHOM composes an international and interdisciplinary working group following a purposive sampling mechanism [48], as exemplified by Obbarius and colleagues [27]. This approach enhances scientific rigor and independence of the ICHOM Standard Set development and aims to include representation across key expertise and perspectives a) clinical, b) methodological (i.e. PRO and health system evaluation), c) geographical, d) socioeconomic terms and e) and patients. The inclusion of patients or patient representatives is mandatory and these members account for up to 25% of all working group members [36]. A typical working group consists of 20–25 members, ranging in size from 12 [30] to 31 members [32].

To coordinate the efforts of the working group, ICHOM assigns a project team that performs literature research and devises proposals for the working group meetings. The project team includes therefore an ICHOM-based project leader with significant experience in the field of PRO and the composition of Standard Sets and a research fellow, typically seconded from an external academic institution and with condition-specific expertise (e.g. in clinical practice and/or epidemiology). The project team organizes meetings, polls and other administrative tasks, but is set up to not exert influence on the decision-making of the working groups that is informed by technical (evidence-based) and lived (experience-based) expertise. Additionally, a project chair, typically a senior clinician with a strong scientific background in the respective field, supports the work with subject-matter expertise and clinical advice. Typically, these clinicians pilot selected PROMs and drafted Standard Sets in their clinical work to provide advisory support for the ongoing development and implementation of the respective Standard Set.

#### Process of evidence collection and structuring

The project team’s core task is to conduct a systematic literature review following the PRISMA guidelines for systematic reviews [49] and the compilation of relevant outcomes, which provides the basis for all Standard Set developments. Typically, case registries are included if possible and working group members are encouraged to identify additional relevant resources. Outcome domains of interest center around a) disease-specific survival and disease control, b) symptoms, c) functioning and quality of life, d) disutility of care and e) healthcare utilization. These domains have been exemplarily outlined by Kim and colleagues [28]. Yet, some working groups adjust the domains to be clinically appropriate [21] for the given condition under review or define different domains for different periods of the cycle of care [17, 18].

To ensure comparability between patient cohorts, however, a standardized set of case-mix variables is included in every Standard Set. Case-mix variables typically cover a) demographics, b) baseline clinical status (e.g. disease stage), c) baseline functional status (e.g. assessment of physical activity or functional scores) and d) treatment factors (e.g. history of surgical treatments for condition of interest).

To reflect the full cycle of care of each condition in the outcomes captured by the respective Standard Set, ICHOM follows a three-tier framework. The principle of the three-tiered approach on covering the entire cycle of care has been discussed by Porter [12]. Generally, the lower tier domains can only be addressed when the higher tier domains are met. The structure of the three-tier framework is, for universal applicability, generic and the primary tier includes domains on the health status achieved or retained, with a focus on survival and measures of the degree of health or recovery at the initial stage. The second tier represents the process of recovery and incorporates measures on functional outcomes and indicators of disutility of care. Lastly, the third tier addresses the sustainability of health and includes measures on the sustainability of the recovery and potential long-term consequences related to the care provided. Notably, the framework suggests a feedback loop back to the first tier to ensure that eventual consequences of the process of care are represented in the outcome measures in the primary (i.e. highest) tier.

Respective measures are selected on the basis of their a) psychometric properties, b) frequency among the patient population of interest to ensure a sufficient coverage of all patients, c) potential impact/relevance of the selected measure on the patient, d) clinical interpretability, and, lastly, e) the feasibility of capturing the measure of interest in a clinical setting. A detailed account on the last criterion is presented in the article by de Roos and colleagues [30]. More recently, working groups (12 out of 27 in total) also performed focus group interviews with patients on the appropriateness of the domains and measures selected in a structured and explicit way before moving to the consensus-building stage of the development process, as outlined below. The integration of the approach is depicted in Fig. 2.

#### Delphi process and the composition of a standard set

Upon completing the collection of available evidence and an optional focus group interview, the working groups convene iteratively for six to eleven remote meetings. After each meeting, they submit anonymous follow-up surveys to build consensus for the domains covered and measures included. A quorum of 75% attendance was defined to account for time zone differences and other organizational issues (e.g. Allori and colleagues [21]). The agenda of the discussion panels is decided jointly by the project team and the working group and evaluates the proposed scope, included domains and specific measures. A modified Delphi process is employed to form consensus among the working group participants to define the core list of outcomes. Although different Delphi approaches have been referenced for the various Standard Sets that have been developed [50–53], the general pattern is similar, requiring agreement on a) the scope, b) the identification of domains and measures, c) their respective inclusion and d) the selection of measurement timepoints. Consensus is achieved when a threshold of 66 to 80% of positive votes is surpassed. To consolidate the core process of decision-making, ICHOM recently proposed a more formalized approach for the Delphi process (Table 2). Votes are casted in the form of an anonymous survey based on a 9-point Likert scale (1 = not important, 5 = somewhat important, 9 = most important), where scores between seven and nine typically represent the equivalent of agreement. Agreement rates between the threshold and 50% lead to a second debate about the item of interest during the next meeting of the working group. All working group participants must endorse the draft in order for it to proceed to the final step of the Standard Set development. Lastly, after internally agreeing on the composition of the Standard Set, some working groups (14 out of 27) submitted their draft for open or expert review. After the successful completion of the review process, the Standard Set is considered finalized and is prepared for dissemination.

**Table 2:** Consolidated Delphi process to be employed in future Standard Sets to homogenize decision making process

The following pass criteria will be used to agree on a minimum Standard Set of the most relevant outcomes	
Domain ranked between 7 and 9^a^ by > = 80% of the working group	Inclusion
Domain ranked between 7 and 9^a^ by < 80% of the working group	Inconclusive^b^
Domain ranked between 1 and 3^a^ by > = 80^a^ of the working group	Exclusion

^a^All rankings are based on a 9-point Likert scale

^b^All inconclusive domains will enter a second round of voting

## Discussion

### The evolution of scope and methodology

Since its establishment in 2012, ICHOM’s scope has evolved from exclusively focusing on developing Standard Sets for conditions with a high disease burden and poorly standardised care towards a more multi-faceted selection approach. This new approach now includes aspects of the clinical care setting and the feasibility of developing a comprehensive, yet condensed Standard Set to facilitate implementation in day-to-day care settings. This underscores ICHOM’s focus on improving the quality of care. Yet, this comes at the cost of conceptual and methodological homogeneity (e.g. a separate Standard Set for different stages of the same condition [15, 16], instead of only one Standard Set for reasonably similar conditions [20, 23]), which requires even closer and more frequent consultations with stakeholders as part of the consensus-based decision-making process. This challenge has been acknowledged elsewhere [13], but constitutes one of the key levers to provide flexibility and broad applicability of the ICHOM methodology.

Similarly, the methodology has evolved from a strong and static emphasis of the tier-based approach by Porter [12, 46] to a more dynamic and flexibly applicable approach: Although the principles of Porter’s work continue to provide the foundation for ICHOM’s Standard Sets development approach, some working groups have developed different approaches to account for the nature of the specific condition. Mahmud and colleagues, for example, assess the domains outlined above along the specific surgical procedure of the condition addressed [17], whereas McNamara and colleagues distinguish between longitudinal and procedure-specific outcome measures when discussing coronary artery disease [18]. Salinas and colleagues, lastly, assess the patient-reported health status mostly via the PROMIS-10 questionnaire [54] and, hence, rely on a validated core set of patient-centered outcomes that is only complemented by selected individual parameters to accentuate the scope of the Standard Set accurately [25]. This emphasizes that ICHOM does not aim to follow a methodological dogma, but promotes a pragmatic approach towards selecting an appropriate methodology that is guided by principles rather than rules. To sustain this approach, however, a core concern of ICHOM is a reliable and reproducible decision-making process for the composition of its Standard Sets and, hence, a more precise outline of the Delphi process methodology has been proposed (Table 2), which is set to become the standard in all future Standard Sets.

### Validation and implementation

For a thorough validation of the proposed Standard Sets, external input, review and feedback proved to be critical. However, the later Standard Sets in particular faced significant challenges in recruiting patient focus groups ex ante. An internal focus group, composed of patients and patient representatives of members of the working group, eventually proved to be helpful and is now becoming the standard approach for early patient input prior to the working group meetings.

Additionally, an open review process was enabled through the distribution of Standard Set drafts to patients and provider organisations. Additionally, post-implementation studies have been published in peer-reviewed scientific journals involving numerous Standard Sets that provide a valuable source of feedback for the continuous improvement of existing Standard Sets and guidelines for future projects: Although the benefits of PRO and the structure provided by the Standard Sets are much acknowledged [55–57], heterogeneity among measures included and increased organizational work pose challenges to the implementation [58, 59]. ICHOM itself conducts ongoing internal reviews and identifies a lack of clinical resources, challenges around the implementation of Standard Sets in existing clinical routines and cost-related issues for the use of proprietary or licensed PROMs as challenges that need to be addressed. Many of these issues have also been highlighted by other researchers who propose the implementation generic sets of PROMs instead of condition-specific sets of PROMs to a) enable the assessment of PRO across different conditions, b) the use in the context of multimorbidity and c) the easy-to-use implementation at the point of care, e.g. through short forms or device-based computerized adaptive tests (CAT) [60]. More recent work has also focused on the compatibility of ICHOM Standard Sets to already established frameworks and highlights the importance of a further homogenisation between proposed PROMs and established patient-reported outcomes measurement information systems (PROMIS) to facilitate the collection of PROMs at the point of care [61]. ICHOM has been acting upon this valuable feedback and has launched a harmonisation initiative that focuses on the consolidation of semantic meaning across core measures, which can be applied across different spectra of conditions without a condition-specific adaptation [14]. This initiative provided the basis for the establishment of an ICHOM taxonomy that eliminates redundancy from existing Standard Sets and enables the interoperable use of Standards Sets in the existing, machine-readable formats SNOMED CT and LOINC [14]. Further efforts also aim at the realignment of PROMs across different countries and healthcare systems, respectively.

### ICHOM’s position in relation to other PRO / functional outcome reporting initiatives

ICHOM considers itself a complement in the growing field of initiatives working towards the patient-centered improvement of outcome reporting and puts a particular emphasis on the measurement of quality in care for a broad spectrum of different conditions. In contrast, other well established initiatives (e.g. OMERACT [45, 62], SONG [43]) cover a more narrow spectrum and apply a much more condition-specific methodology, specifically developed to meet the requirements of rheumatological conditions or clinical manifestations of end-stage renal disease (ESRD), respectively. Additionally, although their focus has been shifting towards care-related issues (e.g. SONG requires outcomes to be measured “in a meaningful, appropriate, and easy way as accurately as possible” [63]), both initiatives greatly emphasize endpoint homogenisation for scientific reporting. Due to the different focus, we do not consider the ICHOM Standard Sets on ESRD by Verberne and colleagues [31] and on inflammatory arthritis by Oude Voshaar and colleagues [36] as redundant, but as a complementing perspective on the care delivered for these specific conditions, providing a quality monitoring tool as compared to a trial reporting tool. We are, however, aware of the fact that the parallel implementation of multiple Standard Sets and with different foci is challenging and most likely not practical in a clinical setting. Additionally, without any particular scope, but with a strong focus on methodology, the COMET initiative [64] has also become a key player in the field of PRO / functional outcome reporting. We see that all of the initiatives above use aspects of the COMET methodology in their own approaches and two ICHOM Standard Sets explicitly state the COMET guidelines as a core component of their methodology [26, 41]. Other governmental initiatives, like the US-based National Quality Forum (NQF) or the Patient-Centered Outcomes Research Institute (PCORI) and the Dutch Programme for Outcome Based Healthcare [65] support efforts for the development of PRO / functional outcome reporting through endorsements of applicable measures and funding, respectively. The widespread interest in PROMs has furthermore led to the exploration of adjacent fields like quality improvement and health technology assessment (HTA) [66]. These efforts underscore, from our perspective, the importance of value-based principles for future developments in patient care, performance assessment and quality benchmarking in modern healthcare systems. Yet, they also highlight the complexity of the field and the demand for further alignment and a focus on the compatibility between the different initiatives and their purposes.

## Conclusion

In the context of unsustainable healthcare spending and sub-optimal value of delivered care, ICHOM has become one of the shaping forces in the field of PRO / functional outcome measurement coupled with clinical outcomes. With its aspiration to provide a broadly applicable approach for a diverse spectrum of medical conditions and care settings, its methodology has evolved over the past decade from a theoretical framework to a repeatedly validated, dynamic methodological approach that facilitates the development of a scientifically valid Standard Sets. Yet, further homogenisation – along the dimensions of conditions covered and measures collected - is required to facilitate the compatibility with other initiatives and the widespread implementation in clinical settings.

## Data Availability

All primary literature used is available as referenced, yet internal process documents are only available upon reasonable request from the corresponding author (L.B., leo.benning@uniklinik-freiburg.de).

## Abbreviations

CAT: Computerized Adaptive Test
CHIP: Children’s Health Insurance Program
COMET: Core outcome measures in effectiveness trials
ESRD: End-stage renal disease
GDP: Gross domestic product
HTA: Health technology assessment
ICHOM: International Consortium for Health Outcomes Measurement
LMIC: Low and middle income countries
NQF: National quality forum
OAB: Overactive bladder syndrome
OMERACT: Outcome measures in Rheumatology
PCORI: Patient-centered outcomes research institute
PRISMA: Preferred reporting items for systematic reviews and meta-analyses
PRO: Patient-reported outcome(s)
PROMIS: Patient-reported outcomes measurement information system
SONG: Standardised outcomes in Nephrology
VBHC: Value-based health care
